# COVID-19 “Nightmare”: Perceived Stress, Emotional Distress, and Burnout Syndrome among Medical Staff after One Year of the COVID-19 Pandemic

**DOI:** 10.3390/jpm13121640

**Published:** 2023-11-24

**Authors:** Maria Victoria Ruta, Teodora Alexescu, Doina Adina Todea, Nicoleta Stefania Motoc, Octavia Luiza Necrelescu, Andrada Urda Campean, Claudia Toma, Catalina Angela Crisan, Milena Adina Man

**Affiliations:** 1 “Leon Daniello” Clinical Hospital of Pulmonology, 400012 Cluj-Napoca, Romania; victoria.suteu@yahoo.com (M.V.R.); viana115@yahoo.ca (O.L.N.); 2 Department of Internal Medicine, Faculty of Medicine, Iuliu Hatieganu University of Medicine and Pharmacy, 400012 Cluj-Napoca, Romania; teodora.alexescu@gmail.com; 3 Department of Medical Sciences—Pulmonology, Faculty of Medicine, Iuliu Hatieganu University of Medicine and Pharmacy, 400012 Cluj-Napoca, Romania; doina_adina@yahoo.com (D.A.T.); manmilenaadina50@yahoo.com (M.A.M.); 4 Department of Biostatistics, Iuliu Hatieganu University of Medicine and Pharmacy, 400012 Cluj-Napoca, Romania; aurda@umfcluj.ro; 5 Faculty of Medicine, Carol Davila University of Medicine and Pharmacy, 020021 Bucharest, Romania; claudiatoma@yahoo.co.uk; 6 Department of Neurosciences, Discipline Psychiatry and Pediatric Psychiatry—Pulmonology, Faculty of Medicine, Iuliu Hatieganu University of Medicine and Pharmacy, 400012 Cluj-Napoca, Romania; ccrisan2004@yahoo.com

**Keywords:** COVID-19, burnout syndrome, emotional distress, disease perception

## Abstract

(1) After one year of treating patients with SARS-CoV-2 infection, physical exhaustion is added to emotional stress and burnout syndrome. (2) By applying specific questionnaires, we evaluated healthcare workers who handled patients infected with SARS-CoV-2in terms of disease perception, perceived stress, emotional distress, and burnout syndrome after one year of the COVID-19 pandemic and compared them with staff who did not handle infected patients. (3) A total of 165 persons were evaluated, with 79 working in the COVID-19 department and 86 working in the non-COVID-19 department. No statistically significant differences were found in the perceived stress scores, emotional distress (functional or dysfunctional), and disease perception among the COVID-19 and non-COVID-19 groups. Also, we did not find any differences between the COVID-19 and non-COVID-19 departments concerning their total Maslach scores—50 in the COVID-19 department and 51 in the non-COVID-19 department, *p* = 0480—so a moderate level of burnout in the two groups. The only statistical difference was in the Maslach depersonalization scores, which were higher among COVID-19 workers (*p* = 0.024). (4) In our center, there was no statistically significant difference in perceived stress or emotional distress. The level of burnout syndrome seems to be the same among the two groups, regardless if they worked withCOVID-19-infected patients or not.

## 1. Introduction

Coronavirus disease 2019 (COVID-19) is an infectious disease that quickly spread around the world, forcing the World Health Organization to declare it a pandemic on 11 mars 2020 [1]. Although humankind has faced numerous pandemics so far (the plague (seventeen century); Cholera (1841–1859); Spanish flu (1918); SARS (2003); H1N1 (2009); Ebola; Zika; and MERS (2014~2016)), through its dynamic, pleomorphic manifestation, lack of specific treatment, and its unpredictable outcome, this one seems to have put people around the world through a hard trial [2,3]. Healthcare workers’ mental health during pandemic times has always been an important issue. There are numerous data that indicate the fact that frontline health professionals experience compromised mental well-being during pandemics. They can manifest as moderate-to-high levels of anxiety, depression, nervousness, and insomnia and, to a lesser degree, stress [4,5,6,7,8,9,10,11,12,13,14,15]. Recognizing and addressing burnout and emotional distress is an important issue in the medical field in general and, in particular, in times like COVID-19, when everything was new. Depending on the country, its culture, the hospital, and the facilities that the hospital provides, the medical staff feels more or less secure and “in good hands”. For example, evaluating the medical staff in our hospital (Cluj Napoca, Romania) in 2020, in the first month after the COVID-19 outbreak, we did not find any statistically significant differences between the medical personnel working with COVID-19 patients versus those not working in the COVID-19 department in terms of perceptions, levels of stress, emotional distress, and coping strategies [9], unlike other authors in different settings where there was a difference between various groups (Spain) [10]. From the significant increase in stress and stigmatization among medical personnel [11] to acute levels of fear, depression, anxiety, and stress [12,13], the medical staff seem to have fewer and fewer resources. During the pandemic, the medical staff went through many stressful situations and experiences that led to their burnout, many of them being related to concerns about their families’ health status, their hospital’s support of their personal needs in case of an infection they developed, and poor communication [14]. According to an online survey, 26% of healthcare workers in the United States have contemplated quitting their jobs since the onset of the pandemic. This includes 14% who have expressed thoughts of potentially leaving the healthcare profession entirely due to the impact of COVID-19 [15]. Another aspect that medical workers must face after this year of COVID-19 is burnout syndrome. It represents the sensation of overwhelming exhaustion, detachment from the job, ineffectiveness, and lack of accomplishment as a result of prolonged exposure to chronic interpersonal stressors on the job. Burnout syndrome affects individuals’ conception of themselves and others [16,17,18]. Although healthcare professionals, particularly in some categories (residents, nurses, surgeons, intensive care units, and emergency department healthcare workers), are prone to developing burnout syndrome sometime during their career, this year, all healthcare workers dealing with COVID-19 have been exposed to an enormous workload due to the important number of patients and lack of personnel, and were either ill, or in search of another career. According to Freudenberger, who was the first to describe burnout syndrome, the individuals who are more susceptible to burnout are those who are dedicated and committed [17,19,20,21].

Considering that, at least at the beginning, the personnel working with SARS-CoV-2 patients did that on a volunteer basis, at least in our hospital, this is more than enough of an argument in favor of burnout. An important point during this last year was the appearance of vaccines with good protection from the disease in general and 100% from severe disease [19].

The physician burnout issue related to electronic health records (EHRs) was examined from 1 January 2016 to 31 January 2021 and it was shown that administrative burden increased burnout. Excessive workflow affects work-life balance and relationships with other colleagues, and it automatically contributes to the loss of control over one’s autonomy and one’s schedule [22].

Our hypothesis was that after one year of working with COVID-19 patients, the levels of stress and burnout syndrome would be much higher among the healthcare workers who dealt directly with confirmed SARS-CoV-2-infected patients.

Therefore, the purpose of the present study is to evaluate disease perception and burnout syndrome after one year of the COVID-19 “nightmare” among healthcare workers in the COVID-19 department versus the non-COVID-19 department. As COVID-19 changed a lot of work-related aspects, we sought to see if the disease was perceived differently among personnel after one year of being exposed to COVID-19 patients as opposed to those who have not been exposed to COVID-19 patients.

## 2. Materials and Methods

The present study was a cross-sectional study consisting of a survey among medical staff from a tertiary pulmonology hospital dealing with COVID-19 patients for one year. We evaluated the medical staff after one year of being exposed to COVID-19 (COVID-19 department) and compared them with the same hospital staff that had not been exposed to COVID-19 patients (non-COVID-19 department). The study took place in a teaching hospital that was on the front line of the battle against COVID-19 from the beginning of the pandemic (March 2020). We decided to analyze the staff from one hospital only because of an internal measure to improve the medical act by increasing the job satisfactionof the staff employed.

Participants included were all medical staff: doctors, junior doctors/residents, nurses, and caregivers (including pharmacists and radiologist technicians) (see Table 1). As we tried to evaluate the impact of the “COVID-19 nightmare”, we called on the same participants that were evaluated at the beginning of the pandemic and applied the questionnaires to the same medical staff. The reasons for choosing only the medical staff from one hospital were the different conditions. Due to the extraordinary conditions, each hospital managed the pandemic in a particular way. The participants were divided into 2 groups according to COVID-19 exposure. The COVID-19 group comprised all healthcare workers from the following departments: the COVID-19 pulmonology ward, ICU department, radiology department, and laboratory personnel, as they dealt directly with confirmed COVID-19 patient samples. The non-COVID-19 group included healthcare workers from the following departments: the pulmonology ward, tuberculosis dispensary, thoracic surgeons, and pharmacy (see Table 2). The work schedule changed several times during this period for COVID-19 personnel. At the beginning of 2020, it was 8 h shifts and 24 h breaks without any free days (no weekends, no holidays), then 12 h shifts with 48 h breaks without any free days or holidays, and since January 2021, it has been 8 h shifts with weekends and holidays. For the non-COVID-19 participants, there were eight-hour shifts, with free weekend days. Personal protective equipment was available without restriction for all participants.

### 2.1. Data Collection

The research investigated the perceptions of illness, stress levels, emotional distress, and burnout levels among hospital employees using well-established questionnaires. It was approved by the Local Hospital Ethics Committee (17/1 July 2023). As the psychological evaluation is a part of the annual medical staff check-up, the questionnaire was filled out during this interview after verbal and written consent. The participation of the employees was voluntary, and the questionnaires were distributed and completed under the supervision of the hospital psychologist. Eachquestionnaire was afterwards verified and blanked, covering the indentity of respondent. This internal study to complete the previously detailed questionnaires took place over a period of 6 months.

The study instrument consisted of four questionnaires: the Brief Illness Perception Questionnaire (IPQ), a Perceived Stress Scale (PSS 10), a Profile of Emotional Distress (PDE), and the Maslach questionnaire to evaluate burnout syndrome. 

The questionnaires were personally completed by each participant on site individually in the following order: PSS-10, PDE, Maslach, and the Brief Illness Perception Questionnaire (IPQ).

PSS 10 [23], PDE [24], and IPQ [25] have been previously described in a paper already published [9]. We will recap it here briefly. 

### 2.2. Perceived Stress Scale (PSS 10)

The PSS 10 is a multiple-choice questionnaire designed to evaluate an individual’s perceived stress levels over the past two weeks. A score falling between 0 and 12 signifies a medium–low level of stress, with no clinical significance attached. Scores between 13 and 19 indicate a medium–high level of stress and may carry clinical significance. A score exceeding 20 signifies a high level of stress and may have clinical significance. It indicates that, in recent weeks, the individual has encountered a substantial number of demanding situations and has either not been able to manage them or has faced them with considerable difficulty. This outcome is typical of individuals dealing with significant stress-related problems [9,21].

### 2.3. Profile of Emotional Distress (PDE)

This questionnaire assesses the presence of negative emotions, encompassing both functional and dysfunctional aspects such as anxiety, worry, depression, and sadness. It consists of 26 adjectives that describe these negative emotions and is categorized into six subscales. This scale allows for the calculation of an overall distress score as well as separate scores for “functional fear”, ”dysfunctional fear”, “functional depression/sadness”, and “dysfunctional depression/sadness”. In this particular study, an overall distress score was computed [9,22].

### 2.4. Maslach Burnout Evaluation Questionnaire

The Maslach Burnout Inventory is the gold standard for evaluating burnout (18). It is a 25-item questionnaire and is structured on 3 dimensions: emotional exhaustion (9 items), depersonalization (6 items), and reduction in personal achievements (10 items). As a way of answering the items, we used a 5-step Likert scale as follows: 1—very rare, 2—rare, 3—sometimes, 4—frequent, and 5—very frequent. The advantage of this scale is that it allows for a greater variety of answers and thus reduces the risk of obtaining the same answer from most subjects. Burnout assessment is performed on a scale with three dimensions labeled as: emotional exhaustion (EE), depersonalization (DP), and cognitions of efficiency and professional achievement. Thus, a high level of burnout involves subjects obtaining high scores on exhaustion subscales (e.g., I feel exhausted from work, I feel I have reached the end of my powers), and depersonalization (e.g., I do not really care about what happens to some of my patients/clients/students).

In order to counteract the effects of a possible monotony in giving the answers, items quoted in reverse were interspersed throughout the questionnaire, with the subjects being “obliged” to pay attention to their formulation (the individual’s need for consistency).

Afterwards, the sum of the points for each dimension was calculated and the total score gave the level of burnout severity. A low level was obtained when the total score ranged from 25 to 50, medium when the score ranged from 51 to 75, and a high level of burnout was when the score was 76–125 [23].

### 2.5. The Brief Illness Perception Questionnaire (IPQ)

This is a nine-item questionnaire that assesses cognitive perceptions of illness, covering areas such as the impact on life, illness duration, personal control, treatment effectiveness, symptoms, illness concern, and understanding of the disease. Each question scored from 0 to 10, with higher scores indicating a more catastrophic perception of the disease. The total score ranged from 0 (least affected) to 80 (most affected).

Participants also completed a supplementary questionnaire about their demographics, health status, social interactions, and more [24,26].

All participants gave their informed consent to complete the questionnaires. According to the authors’ recommendations, all questionnaires were analyzed and interpreted by the same hospital clinical psychologist.

Additionally, all participants filled out an additional questionnaire created by the study’s researchers. This questionnaire collected information about their gender, age, occupation, marital status, educational background, smoking habits, alcohol consumption, other existing health conditions (including psychiatric issues like anxiety and depression), and the extent to which they had recently been able to discuss their problems with someone.

Statistical analyses were conducted utilizing IBM SPSS STATISTICS 25.0 software for Windows (IBM, Chicago, IL, USA). Median values (25th and 75th percentiles) were computed for all scores (ordinal variables) and quantitative variables that did not exhibit a normal distribution. In cases where the data followed a normal distribution, a *t*-test for independent samples was employed, using equal or unequal variances based on the outcome of the Levene test. When the data did not meet this assumption, the Mann–Whitney U test was used for sample comparisons. For qualitative variables, the differences between two frequencies were assessed using the Chi-square test or the Fisher exact test if the conditions for the Chi-square test were not met. A significance level of *p* < 0.05 (two-tailed) was considered to indicate statistical significance.

## 3. Results

A total of 165 of the 212 questionnaires distributed were returned. Apart from the pharmacy staff (6 respondents), who did not complete the IPQ, 138 respondents successfully answered all the items. Of the 144 the respondents, 119 were women. All respondents had a median age of 45, whereas the COVID-19 group employees were younger, with ages ranging from 41 (29.75, 49) to 43.5 (33, 48.75). 

Patients’ demographic characteristics are shown in Table 3, where 91 respondents belonged to the COVID-19 group and 53 belonged to the non-COVID-19 group. 

Psychiatric and physiological comorbidities (%) such as anxiety and depression were frequent among all respondents (45, 31.25%). Comorbidities such as cardiovascular disease (arterial hypertension and stroke), gastrointestinal disease (gastritis, ulcers, and gastroesophageal reflux) allergies, asthma, and diabetes were a little more frequent among the non-COVID-19 personnel. 

### 3.1. Perceived Stress, Emotional Distress, and Disease Perception

No statistically significant difference was found in the perceived stress scores, emotional distress (functional or dysfunctional), and disease perception among the COVID-19 and non-COVID-19 groups (see Table 4).

### 3.2. Burnout Level

We also did not find any differences between the COVID-19 and non-COVID-19 departments concerning their total Maslach scores—50 in the COVID-19 department and 51 in the non-COVID-19 department, *p* = 0480—so a moderate level of burnout in the two groups. 

The only statistical difference was in the Maslach depersonalization scores, which were higher among COVID-19 workers (*p* = 0.024).

## 4. Discussion

The present paper evaluates the levels of perceived stress, disease perception, and emotional distress after one year of COVID-19 among healthcare workers in the COVID-19 and non-COVID-19 departments. It also assesses the level of burnout among healthcare workers after one year of the COVID-19 pandemic. A total of 144 participants were included, with 91 in the COVID-19 department and 53 in the non-COVID19 department. We did not find any statistically significant differences in disease perception, total score of emotional distress, or burnout level in the two groups. The only difference was in the Maslach depersonalization scores, which were higher among COVID-19 workers.

### 4.1. Perceived Stress and Emotional Distress

When analyzing the mental status of health caregivers after the COVID-19 outbreak in 2020 [9], it was not a great surprise that we did not find any statistically significant differences between medical staff that handle COVID-19 patients and those not handling COVID-19 patients. They all had the same levels of emotional distress and perceived stress. It was something new for all medical facilities; everybody was scared and nobody felt that they were “in control”. Even more so, the unpredictable nature of the disease caused problems among medical staff even in areas where COVID-19 was not very prevalent, as shown by Hawari and colleagues [13]. In their paper, they demonstrated high levels of distress in practitioners that were exposed to COVID-19 in an area where COVID-19 did not have a very high prevalence. 

According to more recent data, the prevalence of anxiety and depression among medical staff during the COVID-19 pandemic was higher as compared with other pandemics such as MERS and SARS [25,27,28,29,30].

The abrupt emergence of a life-threatening illness can place an immense amount of pressure on healthcare professionals. This heightened workload, physical strain, isolation, loss of social support, inadequate protective measures, the risk of professional transmission of the virus, and unprecedented ethical dilemmas regarding care allocation can significantly impact their physical and mental well-being [20,27,31].

Repeated exposure to unforeseeable challenges in their line of work can lead to symptoms of anxiety, exhaustion, and stress, commonly referred to as compassion fatigue (CF), burnout, and secondary trauma (ST). On the flip side, there has been an unprecedented surge in public support for healthcare workers, with society rallying around them like never before [21,27,32].

Healthcare workers often experience emotional tiredness, which can result in medical errors, reduced empathy in patient care, decreased productivity, and higher turnover rates. The ability of healthcare professionals to effectively cope with stressors is crucial not only for their patients but also for their families and their own well-being. Healthcare providers exhibit varying levels of psychological resilience, which influences their capacity to adapt positively to adversity and shield themselves from stress. Prior to the COVID-19 pandemic, extensive research had already underscored the multifaceted nature of stressors in healthcare, including responsibilities related to electronic health records, insurance and billing complications, patient dissatisfaction, and the challenge of balancing a demanding work-life schedule [33]. Given the numerous known and unknown consequences of COVID-19, various studies have highlighted the prevalence of stress, fear, anxiety, and symptoms of depression, with a particular focus on frontline healthcare workers [34,35,36,37].

In a comprehensive Chinese survey involving 5062 healthcare workers, Zhou and colleagues [27] found that 29.8% of the participants reported experiencing stress, 24.1% reported anxiety, and 13.5% reported depression. Similarly, another study by Liu et al. [8], which assessed distress, anxiety, and symptoms of depression in 4679 Chinese healthcare workers, revealed that the prevalence of anxiety and distress was approximately 16% each, while 34.6% of respondents reported experiencing symptoms of depression [35]. In our study, perceived stress among healthcare workers was 14, which is a medium/high level of stress with significantly higher values among those who dealt with SARS-CoV-2-infected patients. This is quite natural considering the workload that all healthcare workers have dealt with in the last 12 months. We did not evaluate anxiety and depression separately, but they have been reported by our responders separately at psychiatric disorders in many cases: 12 out of 144 with anxiety and 33 out of 144 with depression. Not surprisingly, psychiatric disorders were more frequent among those that worked in the non-COVID-19 department. The level of perceived stress and emotional distress is even more significant when we compare them with personnel that did not even have contact with COVID-19 patients, such as pharmacists. The hospital comprises two building completely separated from each other, and the pharmacy and laboratory personnel did not have direct contact with COVID-19 patients. This could emphasize the direction in which to intervene to reduce burnout syndrome among COVID-19 personnel by changing unhealthy mindsets. The functionality or dysfunctionality of an emotion is based on personal experiences associated with that emotion, personal beliefs, and behavioral consequences of that emotion. Therefore, this categorization is based on qualitative differences that exist between emotions with the same valence—a difference that is given by the person’s personal beliefs and not by variations in the emotion’s intensity [38].

Disease perception did not differ after one year of SARS-CoV-2 infection experience. It was similar when we compared COVID-19 staff with non-COVID-19 staff or when we conducted the subgroup analysis. There was also no difference between those who received the vaccine and those who did not receive the vaccine. The only thing that was a bit higher among those who had the shot was the IPQ1 score (consequence score—disease impact on one’s life). They either had the disease or not and they hurried to have the vaccine as the consequences imagined by them were disastrous. 

### 4.2. Burnout Syndrome

Burnout syndrome is a frequently observed issue in the career of any medical professional [18,39]. However, it is gaining heightened attention in the current context, particularly among healthcare workers dealing with COVID-19 patients. This increased attention is warranted due to the significant negative consequences associated with burnout, which include depression, an elevated risk of medical errors, and adverse impacts on patient safety [39,40,41].

The recognition of burnout as a serious problem among medical practitioners is on the rise. Several factors, such as years of experience, weekly working hours, weekend work frequency, and team size, may be linked to the prevalence of burnout [20,35,36].

In the present study, the total burnout syndrome score was low/medium (50) in all evaluated HCWs, but when we analyzed COVID-19 versus non-COVID-19, there was no difference. Our results are similar to those reported by Zhang and colleagues, that found a moderate impact of burnout [32], and differ from those published by Wu et al. [34], who reported lower levels of burnout among those who worked with COVID-19 patients compared to those from the “usual ward”. The possible explanation found by the authors was that medical staff who handle infected patients in a controlled manner may feel that they are more in control in terms of controlling some aspects of the disease. Low levels of burnout among COVID-19 HCWs were also reported by Cao et al. [35]. Maunder and colleagues, when analyzing adverse psychological outcomes during the SARS pandemic, reported that intensive care unit and emergency department workers tend to have less general psychological distress and lower burnout scores [36]. This may be because these personnel are used to stressful situations and they are constantly exposed to them, while the HCWs from the “usual ward”, such as the pulmonology ward from our study, are not so much. In some countries, pulmonologists are also a part of the intensive care unit. This is not the case in Romania, even more so in our hospital, where we have intensive care specialists.

In an Asian intensive care unit, high levels of burnout have been documented among nurses and physicians, 52 and 50.3%, results that are similar to ours [37,42]. It was found that the medical staff has a comparable risk of burnout with nurses, as well as comparable mean scores for burnout and indiviaul achievement. These results are consistent with thefindings of Kunz M., Strasser M., and Hasan A. 2019, which showed comparable levels of burnout and general stress for nurses andphysicians [38]. Inour study, there were no higher levels of burnout if we compared the nurses and junior doctors with senior doctors. We believe that the results, being inconsistent with another study conducted in Romania which identified that resident doctors had the highest prevalence compared to the rest of the staff associated with the highest job demands and the lowest job control [39], show that this difference comes from the fact that young doctors in our hospital did not take night shifts and kept their social life as long as possible.

Even more so when analyzing the impact of burnout after SARS or MERS (Batra K study) in the long run, they noticed that 1 to 3 years later, HCWs experienced ongoing high levels of depression symptoms [41]. Our responders had social support, primarily relying on support from their families, followed by support from friends, as reported by Bridgeman and al. Social support, even through technological means, is important as it contributes to mental health [9,43].

The current study comes with several notable limitations. First and foremost, it was conducted in a single center, with participants exclusively from a hospital in Cluj Napoca. Consequently, the applicability of the findings to other populations remains subject to verification. Second, the use of self-administered questionnaires had inherent drawbacks, which could potentially limit the depth of the insights gained. However, despite these limitations, the study provided valuable insights into the psychological well-being of healthcare workers who transitioned from a “typical ward” to a “frontline ward” overnight, where they were tasked with caring for critically unstable patients. Moreover, the study aimed to underscore the pandemic’s far-reaching impact across all sectors of a hospital, including administration.

## 5. Conclusions

Perceived stress and emotional distress (dysfunctional sadness and fear) seem to be higher among persons working in the COVID-19 department compared to the other workers in the same hospital. Disease perception does not differ among the two groups and has not been influenced by vaccination. Burnout is higher among those who have worked with COVID-19 patients than those who have not.

## Figures and Tables

**Table 1:** All healthcare workers.

Profession	COVID-19 (n)	Non-COVID-19 (n)	Total (n)
Doctor	13	8	21
Junior doctor	13	12	25
Nurse	41	19	60
Health caregiver	24	14	38
Total	91	53	144

**Table 2:** All health departments.

Department	COVID-19 (n)	Non-COVID-19 (n)	Total (n)
Intensive care unit	27		27
COVID-19	43		43
Radiology	7	3	10
Tuberculosis		6	6
Pharmacy		6	6
Laboratory	14		14
Non-COVID-19 pulmonology ward		38	38
Total	91	53	144

**Table 3:** Participants (medical staff) demographic characteristics.

Criteria		Total No (%)	COVID-19 No (%)	Non-COVID-19 No (%)	*p*
Gender	Male, no (%)	25 (17.37)	17 (18.69)	8 (15.1)	0.584
Marital status	Married	86 (59.73)	57 (62.64)	29 (54.72)	0.350
	Re-married	3 (2.09)	3 (3.3)	0(0)	
	Divorced	12 (8.34)	6 (6.6)	6 (11.33)	
	Separated	1 (0.7)	1 (1.1)	(0)	
	Single	31 (21.53)	18 (19.79)	13 (24.53)	
	Widow	3 (2.09)	2 (2.2)	1 (1.89)	
	In a relationship	4 (2.78)	2 (2.2)	2 (3.78)	
School	Secondary school	3 (2.09)	2 (2.2)	1 (1.89)	
	Highschool	16 (11.12)	10 (10.99)	6 (11.33)	
	Nursing school	51 (35.42)	40 (43.96)	11 (20.76)	0.003
	University	74 (51.39)	39 (42.86)	35 (66.04)	
Profession	Doctor	21 (14.59)	13 (14.29)	8 (15.1)	0.563
	Junior doctor	25 (17.37)	13 (14.29)	12 (22.65)	
	Nurse	60 (41.67)	41 (45.06)	19 (35.85)	
	Health caregiver	38 (26.39)	24 (26.38)	14 (26.42)	
Smoking status	Active smoker	46 (31.95)	35 (38.47)	11 (20.76)	0.088
	Former smoker	17 (11.81)	10 (10.99)	7 (13.21)	
	Non-smoker	81 (56.25)	46 (50.55)	35 (66.04)	
Alcohol consumption	Yes	2 (1.39)	2 (2.2)	(0)	0.711
	No	59 (40.98)	36 (39.57)	23 (43.4)	
	Occasionally	78 (54.17)	50 (54.95)	28 (52.84)	
Psychiatric and physiological comorbidities (%)	Anxiety	12 (8.34)	7 (7.7)	5 (9.44)	0.760
	Depression	33 (22.92)	21 (23.08)	12 (22.65)	0.952
	Difficulty concentrating	49 (34.03)	27 (29.68)	22 (41.51)	0.148
	Others	4 (2.78)	3 (3.3)	1 (1.89)	>0.05
Psychiatric and physiological comorbidities (%)	Yes	73 (50.7)	42 (46.16)	31 (58.5)	0.153
Comorbidities other than psychiatric	Yes	16 (11.12)	9 (9.9)	7 (13.21)	0.541
Social support	None	9 (6.25)	5 (5.5)	4 (7.55)	0.650
	To a small extent	25 (17.37)	16 (17.59)	9 (16.99)	
	Fairly	45 (31.25)	25 (27.48)	20 (37.74)	
	To a large extent	55 (38.2)	37 (40.66)	18 (33.97)	
Vaccination	Yes	98 (68.06)	57 (62.64)	41 (77.36)	0.182
COVID-19 test	Yes	40 (27.78)	29 (31.87)	11 (20.76)	0.151

**Table 4:** PSS-10, PDE, Maslach, and IPQ scores among groups.

	COVID-19 Median (Q1–Q3)	Non-COVID-19 Median (Q1–Q3)	*p*-Value
Age	41 (30–49)	43.5 (33–48.5)	0.691 ^b^
Days from positive PCR test	90 (70–118)	84 (50–90)	0.142 ^a^
PSS-10	14 (10–19)	14 (10–20)	0.840 ^a^
PDE	41 (33–55)	45 (33–58)	0.710 ^b^
Functional sadness	11 (7–14)	12 (8–16)	0.339 ^b^
Dysfunctional sadness	9 (8–13)	9 (8–14)	0.694 ^b^
Functional fear	13 (10–16)	13 (9–18)	0.976 ^a^
Dysfunctional fear	9 (7–12)	9 (7–12)	0.883 ^b^
Maslach emotional exhaustion	21 (15–28)	19.5 (15–24.5)	0.281 ^a^
Maslach depersonalization	10 (8–13)	10 (7–11.5)	0.024 ^a^
Maslach professional realization	20 (16–24)	22 (16–25)	0.195 ^b^
Maslach total score	50 (42–62)	51 (40.5–59)	0.480 ^a^
IPQ 1	3 (2–6)	5 (2–6.5)	0.293 ^a^
IPQ2	3 (1–5)	4 (2–5)	0.162 ^a^
IPQ3	7 (4–8.5)	7 (4–8)	0.809 ^b^
IPQ4	8 (5–10)	7 (4–9)	0.169 ^a^
IPQ 5	3 (1–5)	3 (2–5)	0.338 ^a^
IPQ 6	5 (3–8.5)	5 (3–8)	0.917 ^b^
IPQ 7	7 (4.5–9)	7 (5–9)	0.840 ^a^
IPQ 8	5 (2–7)	4 (2.5–6)	0.848 ^b^
IPQ total	31 (24.5–38.5)	36 (25.5–41)	0.434 ^a^

^a^—*t*-test; ^b^—Man Whitney test; PSS-10—Perceived Stress Scale; PDE—Profile of Emotional Distress; IPQ—Brief Illness Perception Questionnaire. IPQ 1–8 are the responses to each individual question from the questionnaire.

## Data Availability

All data are available with the authors upon request.
